# How to improve pedestrians' trust in automated vehicles: new road infrastructure, external human–machine interface with anthropomorphism, or conventional road signaling?

**DOI:** 10.3389/fpsyg.2023.1129341

**Published:** 2023-05-05

**Authors:** Flavie Bonneviot, Stéphanie Coeugnet, Eric Brangier

**Affiliations:** ^1^Perseus Laboratory, University of Lorraine, Metz, France; ^2^VEDECOM Institute, Versailles, France

**Keywords:** automated vehicle, pedestrian, trust, external human-machine interface (eHMI), anthropomorphism, infrastructure

## Abstract

**Introduction:**

Automated vehicles need to gain the trust of all road users in order to be accepted. To make technology trustworthy, automated vehicles must transmit crucial information to pedestrians through a human-machine interface, allowing pedestrians to accurately predict and act on their next behavior. However, the unsolved core issue in the field of vehicle automation is to know how to successfully communicate with pedestrians in a way that is efficient, comfortable, and easy to understand. This study investigated the impact of three human-machine interfaces specifically designed for pedestrians' trust during the street crossing in front of an automated vehicle. The interfaces used different communication channels to interact with pedestrians, i.e., through a new road infrastructure, an external human-machine interface with anthropomorphism, or with conventional road signaling.

**Methods:**

Mentally projected in standard and non-standard use cases of human-machine interfaces, 731 participants reported their feelings and behavior through an online survey.

**Results:**

Results showed that human-machine interfaces were efficient to improve trust and willingness to cross the street in front of automated vehicles. Among external human-machine interfaces, anthropomorphic features showed significant advantages in comparison with conventional road signals to induce pedestrians' trust and safer crossing behaviors. More than the external human-machine interfaces, findings highlighted the efficiency of the trust-based road infrastructure on the global street crossing experience of pedestrians with automated vehicles.

**Discussion:**

All of these findings support trust-centered design to anticipate and build safe and satisfying human-machine interactions.

## 1. Introduction

### 1.1. Pedestrians' trust in automated vehicles

Automated vehicles (AVs) have the potential to help society by improving safety on roads, saving lives, and lowering fuel use and environmental pollution (Jayaraman et al., 2019; Tafidis et al., 2022). The complete automation of driving is more than just the replacement of drivers by sophisticated technological systems; it is also a global transformation of road use that affects pedestrians. AVs need to gain the trust of all road users in order to be accepted (Habibovic et al., 2018; Bel and Coeugnet, 2023). Concerning pedestrians specifically, the classic beacons allowing crossing must be recomposed, designed, and imagined to ensure a high level of trust in the AVs. Indeed, trust is described as one of the keys to the relationship between humans and technology (Klien et al., 2004). While a few research studies have served as a reference for many studies (e.g., Lee and See, 2004), the definition and understanding of trust remain a matter of debate, including in human–computer interaction. It can be understood as a feeling, a set of beliefs, expectations, or even a motivational state at times. However, trust seems to be multidimensional, based on affective, cognitive, and social dimensions (Karsenty, 2011). Researchers agreed that a low level of distrust would correspond to a high level of trust, thus placing the two concepts on the same continuum (Rotter, 1980; Siegrist et al., 2005). However, trust and distrust are positive and negative valence concepts (Kahneman and Tversky, 1979), with different neuronal functioning (Dimoka, 2010) which suggested that they are distinct but dependent (Bonneviot, 2022).

Concerning specifically trust in automation, Hoff and Bashir (2015) have proposed a well-used model. The strength of this model is that it provides an understanding of the evolution of trust (Muir, 1994; Lee and See, 2004). It is also easily transferable to an automated driving situation (i.e., Habibovic et al., 2018; Holländer et al., 2019; Hjemly and Alsos, 2021), but some adjustments are required when considering an interaction between a pedestrian and an AV (Bonneviot, 2022). However, the operation of AVs relies on explicit communication from other vehicles to interact with their environment (e.g., Düring and Pascheka, 2014), thus neglecting more subtle social cues such as drivers' attention or mood. Research has shown that this lack of understanding of social interactions leads to accidents (Factor et al., 2007) and surprising behaviors that are considered erratic by pedestrians (Habibovic et al., 2013). These feelings affect how people perceive technology and their likelihood to adopt it. It becomes essential to design technologies in a way that people feel comfortable with them (Mori et al., 2012). To ensure safe and effective interaction with technology, consideration of the role of the human in its design is, therefore, paramount (Lee et al., 2015).

Road user interactions take place in a complex social context where norms of use are constructed and reassessed. Non-verbal communication with the driver can defuse ambiguous interaction situations, facilitate decision-making, and increase pedestrian trust in the street crossing situation (Cœugnet et al., 2019). The trust would improve the willingness to interact with technology (Lee and See, 2004), and thus it can be an appropriate way to conceive and ensure pedestrian-automated vehicle interaction. Designing for trust is designing how the technology will communicate with its environment; its external features are key features to ensuring users' trust (Hjemly and Alsos, 2021). One of the main issues in the field of AV, regarding the interaction with pedestrians, is knowing how to successfully communicate with them in a way that is efficient, comfortable, and easy to understand. Some types of communication could influence behaviors, under certain conditions and depending on the channel used (Ackermann et al., 2019). Therefore, automated vehicles need to transmit information to pedestrians through a human–machine interface, so that pedestrians can accurately predict their next behavior and act. As a bridge of communication, the study of human–machine interfaces for AVs is of great significance.

### 1.2. Improving trust through communication

#### 1.2.1. External human–machine interfaces

Several recent research studies have advocated explicit communication through external human–machine interfaces (eHMIs) built into the vehicle (see Rouchitsas and Alm, 2019; Dey et al., 2020). The presence of an eHMI on the AVs would result in a 38% improvement in conflict resolution (Matthews et al., 2017). Pedestrians would also be more willing to cross the street before the vehicle comes to a complete stop (Lagstrom and Malmsten Lundgren, 2015). Pedestrians also report feeling safer (De Clercq et al., 2019) and more confident when the automated vehicle communicates *via* an eHMI (e.g., Sadeghian et al., 2020; Colley et al., 2022). The eHMI would provide a more satisfactory crossing experience (He et al., 2021). Current research also shows that this trust increased with each interaction (Habibovic et al., 2018; Faas et al., 2021). Nevertheless, some studies question the real effectiveness of eHMIs (Métayer and Coeugnet, 2021). For example, Schieben et al. (2019) conclude that the infrastructure plays an essential role in the decision to cross in front of an AV, and eHMI is only useful in situations without road infrastructure (e.g., traffic lights and pedestrian crossing). Furthermore, eHMIs are not always designed with a user-centered approach (Florentine et al., 2015; Clamann et al., 2017). Dey et al. (2020) note that while safety and user experience data were often collected, the effectiveness of the eHMI in facilitating pedestrian crossing was largely ignored in evaluations. Furthermore, the majority of HMI prototypes used only one modality, usually visual, which limits their accessibility for road users with special needs such as the hearing impaired (Dey et al., 2020) or children who need to refer to something they know and thus understand (e.g., Beran et al., 2011).

#### 1.2.2. Anthropomorphic communication

Other modes of communication based on anthropomorphism have been proposed (e.g., Matthews et al., 2017). Studies in the field of AVs are also interested in anthropomorphic headlights that behave like eyes in tracking pedestrian movements (Mahadevan et al., 2018). For example, the Swedish company, Semcom, uses an anthropomorphic design approach on the radiator grille of the car to present a lighted smile as an eHMI (e.g., Yang and OuYang, 2022). According to studies, the more machines resemble humans, the more concerned humans are about their appearance (i.e., called the Uncanny Valley; Mori et al., 2012). In other words, when a robot is designed with human characteristics to perform social interactions, he can cause rejection by generating a feeling of strangeness in the user when he perceives a distance from a man (e.g., in his voice, the features of his face, or the fluidity of his movements; Duffy, 2003). In contrast, Madhavan and Wiegmann (2007) have shown that an anthropomorphic design of HMIs elicits similar responses to those generated in interpersonal social contexts, including trusting attitudes in the human operator. Indeed, several studies have shown that when individuals interact with technology, they apply the social rules of human–human interaction to machines (e.g., Muir and Moray, 1996; Jian et al., 2000), a phenomenon known as ethopoeia (Nass and Moon, 2000). Nass et al. (1996) found that in human–machine interaction, individuals readily formed teams with computers and used socially acceptable behavior such as politeness (Nass et al., 1999). These behaviors observed in human–computer interaction are considered comparable to the trusting behaviors observed in human–human relationships (Muir, 1994). Parasuraman and Riley (1997) justified this phenomenon by saying that trust in automation would embody trust in the designers of automation. Thus, using anthropomorphism for AV communication systems could be relevant to improve pedestrians' trust (Niu et al., 2018) but its appearance should be identifiable as non-human unambiguously. Unfortunately, to our knowledge, no study has tested a user-centered anthropomorphic interface within an AV.

#### 1.2.3. Reinforced communication through infrastructure

Several studies highlight that pedestrians rely mainly on implicit determinants such as changes in vehicle movement when deciding to cross (Clamann et al., 2017; Dey and Terken, 2017; Zimmermann and Wettach, 2017; Dommes et al., 2021). Numerous studies have highlighted that vehicle speed and distance to the pedestrian are effective in influencing pedestrians' decision to cross the street in front of an AV (Beggiato et al., 2017; Dey and Terken, 2017; Bazilinskyy et al., 2019). Zimmermann and Wettach (2017) found that when stopping or accelerating abruptly to indicate the intention to yield, pedestrians experienced negative emotions and little confidence in AVs. In a similar study, Jayaraman et al. (2019) also point out that aggressive driving decreases pedestrians' confidence in AVs but that the presence of traffic lights moderates this effect. This suggests that two sources of information, the AV's behavior and the road infrastructure (e.g., traffic lights), would co-influence pedestrians' confidence in the AV. This could be because pedestrians believe that AVs will be obliquely programmed to respect traffic rules and in particular pedestrian crossings (Meeder et al., 2017). Thus, infrastructure and its organization play a role in pedestrian confidence in crossing the street. As little explored by automation research, infrastructure could be relevant as an avenue to increase pedestrian confidence. Recent projects have developed smart roads connected to vehicles to communicate with pedestrians. Through various visual effects at the pedestrian crossing, the roadway informed pedestrians of dangers or that crossing was possible (Sieß et al., 2015a). Other concepts used lighting. For example, the arrival of the vehicle triggered a light signal at the pedestrian crossing (Siva et al., 2015) or changes in light color temperature could encourage pedestrians to use certain city streets (Sieß et al., 2015b). The Rosegaarde Studio (2017) proposed a luminescent pavement that could improve the visibility of pavements and pedestrian crossings at night. Umbrellium has developed an interactive LED street with a variety of signals, including a pedestrian crossing (Mairs, 2017). These devices, while relevant, have not been designed with users in mind and/or tested with an experimental approach.

### 1.3. Aims and hypothesis

Therefore, the main contribution of this study for future research is to assess the user experience (e.g., utility, usability, satisfaction, and the intention of use) of three different types of HMIs on AV. The last originality of this research is that it assesses HMIs not only in a normed crossing situation but also in a non-normed one, which is rarely studied in the literature. The first communication system was composed of an LED system on the AV (LED eHMI). The second system was based on anthropomorphic communication (i.e., like an onboard vehicle driver). Finally, the third communication system was included in the road infrastructure. All these systems were specifically designed to improve the trust in AVs in a user-centered approach (see Bonneviot, 2022).

Based on the literature review previously presented, the study aimed to test hypotheses. First, we hypothesized that an HMI specifically designed to promote trust in AVs would enhance pedestrians' trust in a street crossing situation compared to an AV without HMI (H1a). In addition, these trust-based HMIs would decrease pedestrians' distrust in a street crossing situation compared to an AV without HMI (H1b). Furthermore, we suggest that an anthropomorphic HMI will increase pedestrians' trust to cross in front of an AV relatively more than an HMI designed with more conventional road signaling (i.e., LED eHMI; H2). To open up a new perspective, we assumed that a communicative road infrastructure will be more efficient to increase pedestrians' trust than an HMI integrated into the automated vehicle (H3a). More than trust, it could be all the user's experience of street-crossing, i.e., perceived safety, trust, distrust, uncertainty, anticipation, willingness to cross, and normed and non-normed crossing behaviors, that could be impacted by these HMIs (H3b). In our study, a better pedestrian experience with a vehicle would correspond to an improvement in its perceived safety, trust, anticipation, willingness to cross, normed crossing behaviors, and also with a decrease in distrust, uncertainty, and non-normed street crossing (i.e., away from pedestrian crossing or in dual-task). Finally, depending on the HMI, participants' trust and willingness to cross may also be different if they are confronted in a standard or a non-standard situation (i.e., riskier, no nominal; H4).

To achieve this goal, 731 participants completed an online survey to estimate their level of trust, their feelings, and their behaviors when crossing the street in front of an automated vehicle associated or not with the three different types of HMIs. The degree of safety was also taken into account in a comparative analysis.

## 2. Method

### 2.1. Participants

A total of 731 participants (52.4% female; 52.8% of 18 to 42 years old; mean = 33.10, SD = 9.4; 47.2% of 43–65 years old; mean = 51.05, SD = 8.56) completed the online survey in an average of 25 min (min = 12.0, max = 103.0, mean = 24.55, SD = 11.20). They received vouchers from the panelist who recruited them.

### 2.2. Tested communication systems

The design of the HMI interfaces was based on a user-centric design approach involving experts in the field of design, engineering, and human–computer interaction (see Bonneviot, 2022). Based on a collection of needs in 42 individual interviews, these interfaces were developed in an iterative process of five creativity sessions, in groups and individually, with researchers in the design and human and social sciences, all from the field of automated mobility. The needs analysis showed four main categories: decision support, physical and legal protection, social interaction, and wellbeing (see Table 1). The focus was on designing interfaces using audio and visual modalities to communicate the intentions of a fully automated vehicle to pedestrians. He et al. (2021) have shown that an audio–visual HMI improved more pedestrians' crossing experience in front of an AV than a single modality HMI. The three HMIs compared in this study promoted distinct ways of interacting with the automated vehicle as discussed previously in the introduction section.

**Table 1:** Pedestrians' needs collected during individual interviews to influence the trust in automated vehicles and that were used to develop the HMIs of the study.

**Pedestrians' needs categories to influence the trust in automated vehicles**	**Verbatim**
Decision support	“At the level of the road environment, a kind of road screen where the main markings such as stop signs, red lights, and pedestrian crossings could change and adapt according to the people waiting at the edge of the road and the traffic. They would change shapes and colors above all, and why not a haptic feedback system, like screens that vibrate at touch. For example for pedestrians, if it is not the moment to cross, the road would vibrate to say that you must quickly remove your foot. For the shape change, I imagined a marking that changes depending on the situation: if a stop line is crossed, the entire road turns red.” (P39)
Physical and legal protection	“There should be a device, at the level of the infrastructures, which can take control of the autonomous vehicle. This device would be able to detect the pedestrian, to identify that the autonomous vehicle has not spotted the pedestrian and suddenly takes control of the autonomous vehicle by making it stop.” (P33) “Create a status in the law, of responsibility in relation to the use of the autonomous vehicle. The pedestrian would be legally protected from the autonomous vehicle.” (P10)
Social interaction	“I would like an emoji in front of the car, as if the car were a person. For example, to be able to personalize her car, whether she is not an object. “I saw you, I saw the zebra crossing, I'm stopping” and a smile. It will also amuse young people, they will laugh. For secure, to reassure them and what's more, it's funny. To reassure that it's not an empty car. We will not see the passenger in front so it shows that it is the driver anyway, but by intermediaries. A smiley and the hand mean stop…an intermediary between humans and a machine.” (P22)
Well-being	“We could imagine that the car broadcasts well-known songs from the Rolling Stones, Michael Jackson or the sounds of birds, it will be much more pleasant in terms of sound.” (P25)

#### 2.2.1. LED eHMI

**Figure 1:**
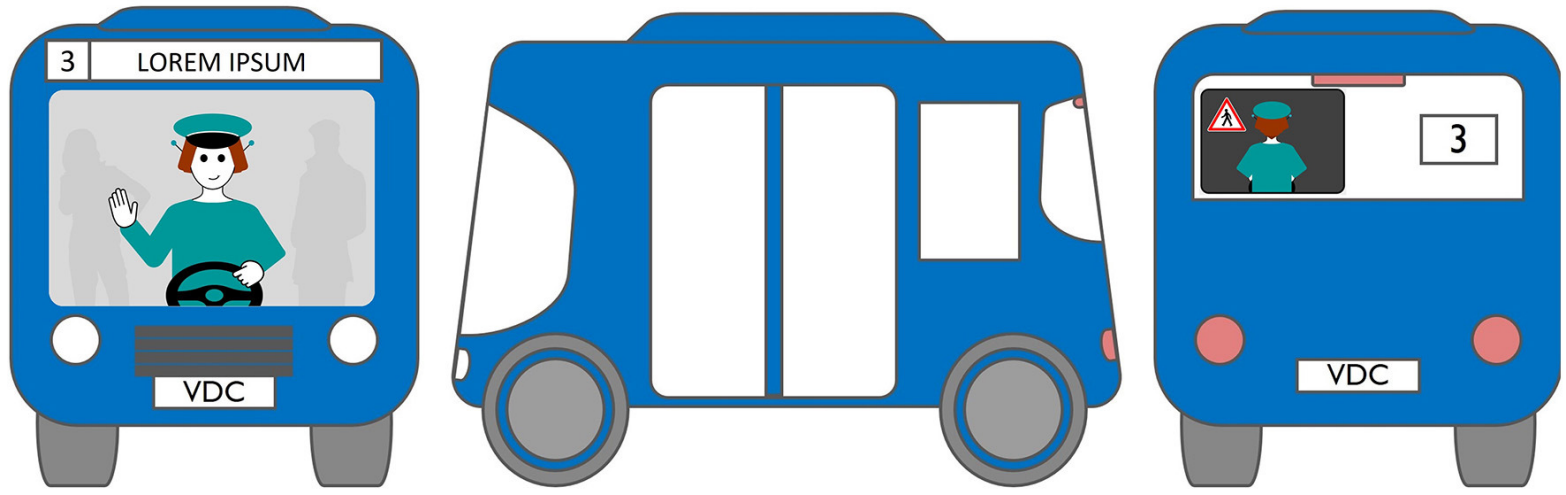
Communication with pedestrians by LED eHMI (“BOLD”), meaning “Pedestrian in sight”.

The first interface, called BOLD (for Bands Of LED), consisted of three LED bands positioned horizontally upper and lower edges of the AV's windshield and at the level of the grille (Figure 1). This was communicating conventional road signaling such as safe crossing (green and white bidirectional arrows with pedestrian symbols), starting (orange halos and a countdown), circulating (moving blue halos), and slowing because of a pedestrian (moving blue halos with a white pedestrian symbol).

#### 2.2.2. Anthropomorphic eHMI

**Figure 2:**
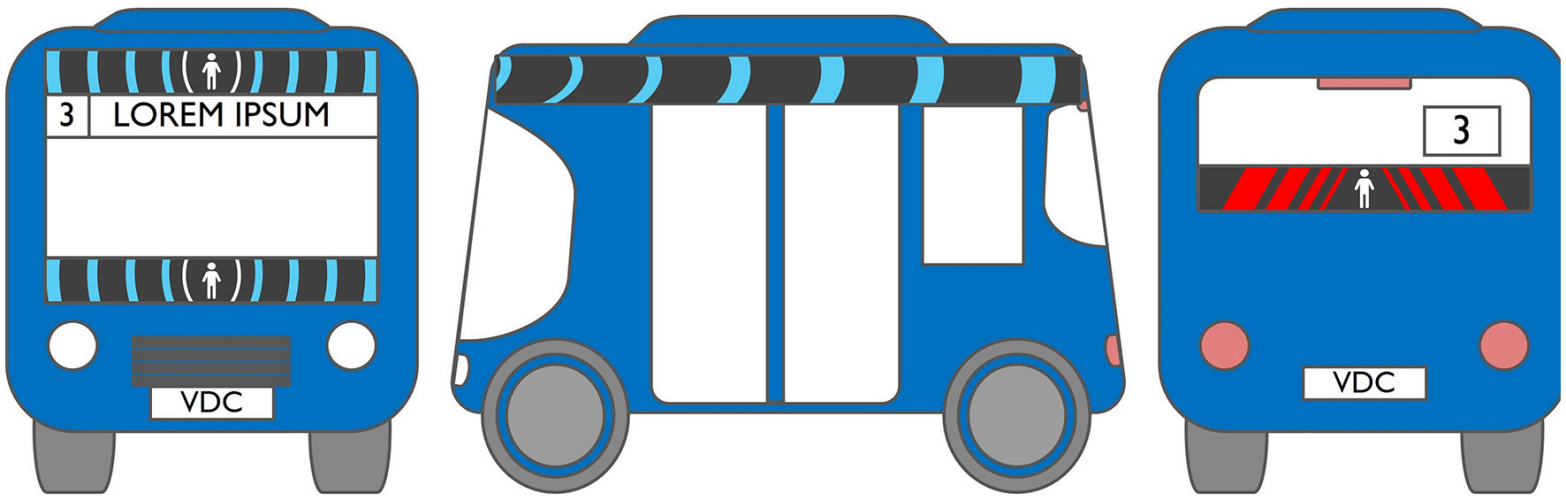
Communication with pedestrians by anthropomorphic eHMI (“Alfy”) meaning “Pedestrian in sight”.

The second external HMI, called Alfy (short for Alfred), had complete anthropomorphic features. It was an avatar of a professional bus driver displayed transparently on the AV's windshield, and at the top left of the rear window was a screen for other road users (see Figure 2). Alfy spoke in a male human voice, it was wearing a cap like a private driver and his clothes changed color for a double coding of messages to improve their identification and understanding. Its messages were: safe crossing (green clothes, open arms, and smiling), starting (orange to red clothes with a neutral face, crossed arms, and a countdown), circulating (blue clothes and hands on the steering wheel), and slowing because of a pedestrian (blue clothes, smiling with a hand salute: see Figure 2).

#### 2.2.3. Communication systems on the road infrastructure

**Figure 3:**
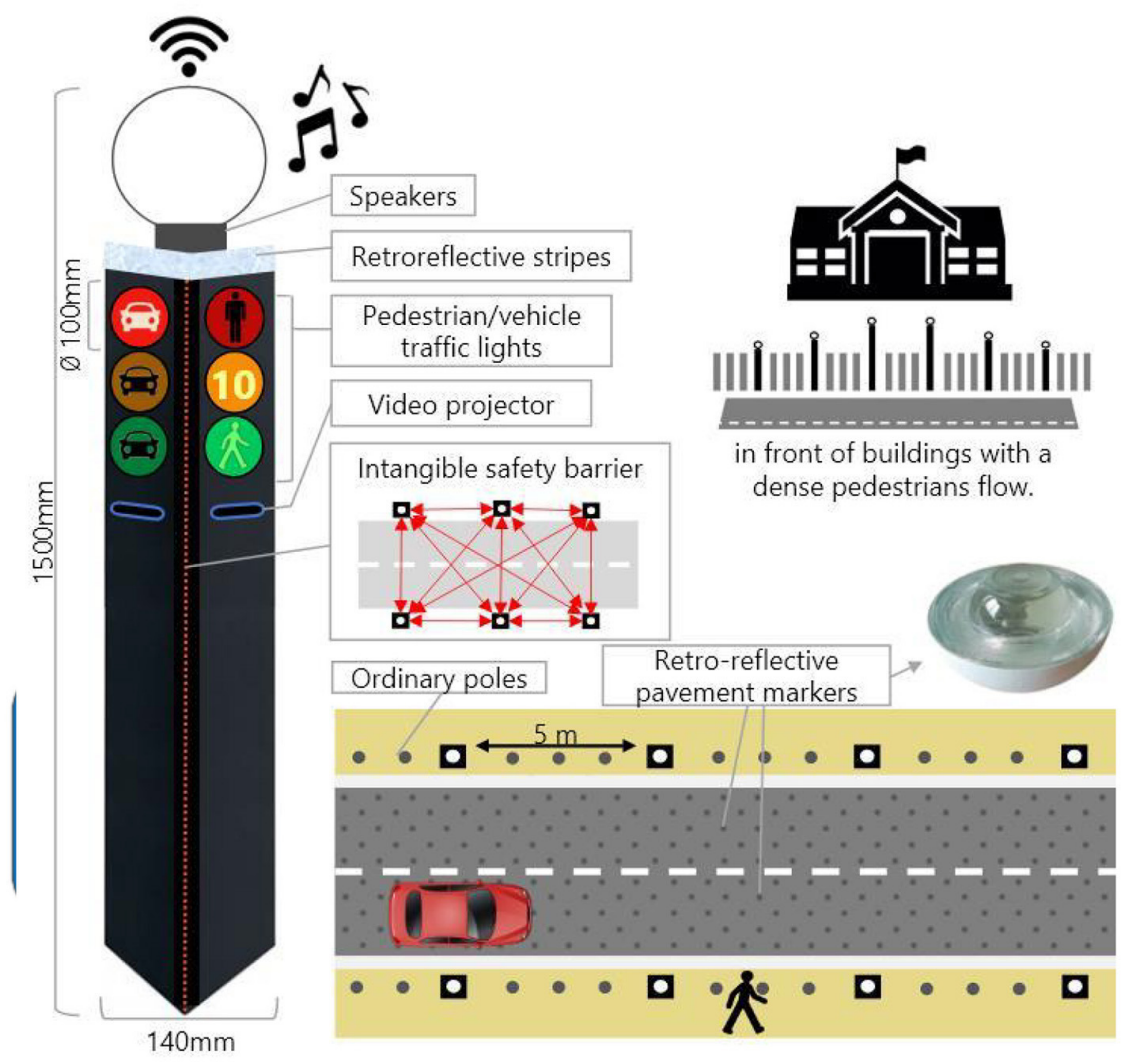
Communication with pedestrians by road infrastructure system (“Sirocco”).

The latter, called Sirocco, was a set of rectangular poles positioned every 5 meters along the roadway and connected to automated vehicles (Figure 3). Those poles were equipped with two-way traffic lights with countdowns for pedestrians and vehicles. Their detectors allowed them to determine the precise position of pedestrians, but especially vehicles, to assess their stopping distance and possibly stop them in the event of a problem. The spacing between poles corresponded to the international regulatory stopping distance before a pedestrian crossing. It is also two times the minimum width of a pedestrian crossing (>2.5 meters) and greater than the maximum length of the longest personal vehicles, such as SUVs (< 5 meters). The Sirocco was equipped with speakers, retroreflective strips to be spotted at night, and video projectors to draw colored patterns on the road due to retroreflective pavement markers. In front of buildings with a strong pedestrian influence, the Sirocco is progressively higher to be better identified by pedestrians and vehicles as a high flux crossing area.

### 2.3. Material

#### 2.3.1. Videos of the communication systems and tested use-cases

Two short videos, based on a sequence of images, were used to present HMIs' usage. The videos consisted of a schema of the road interaction situation, an audio–visual representation of the HMI's-related reaction, and a descriptive text read aloud (Supplementary Videos 1–6). A standard and a non-standard situation were shown to participants. The first standard use case presented interfaces when the vehicle stops at the pedestrian crossing and the pedestrian can cross safely (Supplementary Videos 1, 3, 5). During this video, BOLD and Alfy showed the following message sequence: switching on, starting, driving, pedestrian in sight, safe crossing, starting, and driving (see Figure 2 and Table 2). For the road infrastructure, Sirocco, the video showed the nominal situation, the detection of the pedestrian, the crossing, the end of the crossing, and the nominal situation with the departure of the vehicles (Table 3). Because of its innovative concept, a short description of the Sirocco was added at the beginning of its video. The videos lasted between 90 and 135 seconds. The second situation was a non-standard use case, which corresponded to a dangerous street crossing: as a pedestrian crossed the street in front of a stopped car, a second car overtook, causing immediate endanger to the pedestrian (Supplementary Videos 2, 4, 6). The video was composed of only two alternating frames to give a red flashing effect to certain visual elements. These were two flashing danger signs for Alfy, a flashing red background with a fixed danger sign for the BOLD and a flashing red of the entire portion of the roadway where the pedestrian is located for Sirocco (Table 4). The Alfy, BOLD, and Sirocco videos lasted 31s, 17s, and 11s, respectively.

**Table 2:** Representations of messages displayed by the external human–machine interfaces BOLD.

**Messages**	**Front**	**Sides**	**Back**
Switching on	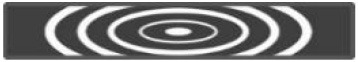	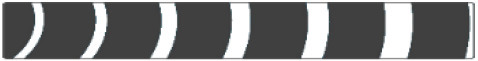	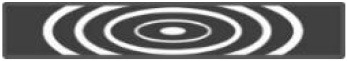
Starting	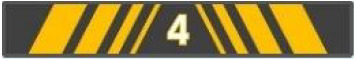	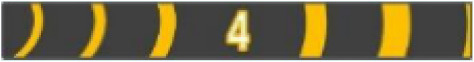	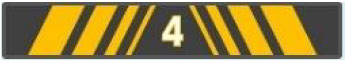 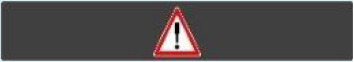
Driving	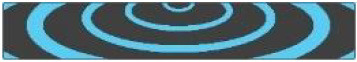	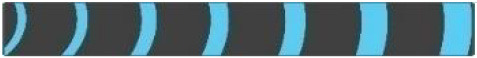	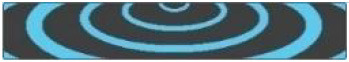
Pedestrian in sight	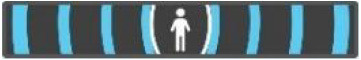	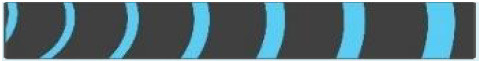	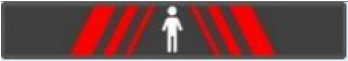
Safe crossing	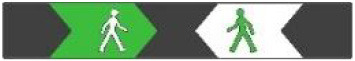	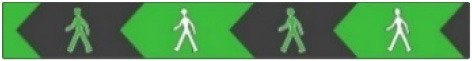	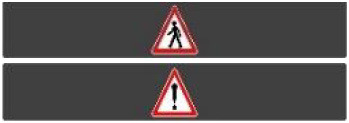
Unsafe crossing	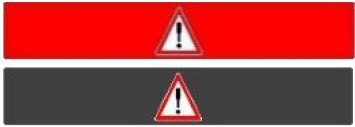	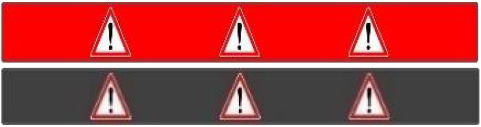	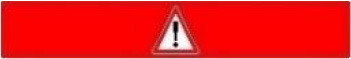

**Table 3:** Representations of messages displayed by the external human–machine interfaces ALFY.

**Messages**	**Front**	**Back**
Switching on	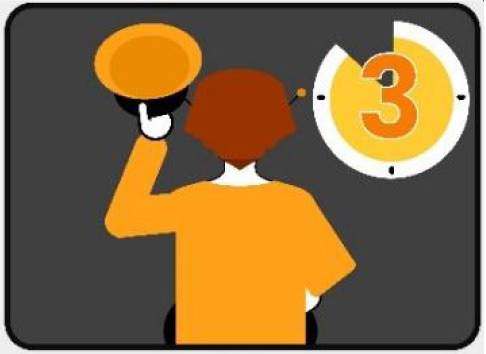	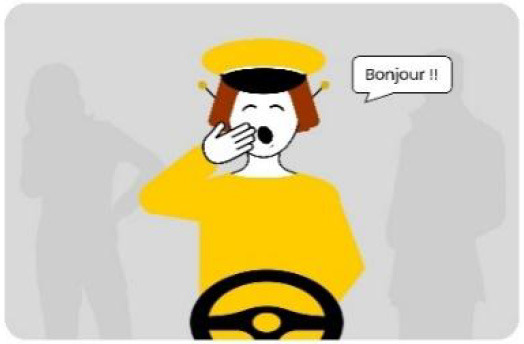
	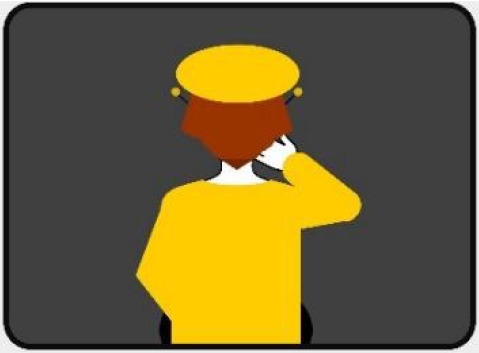	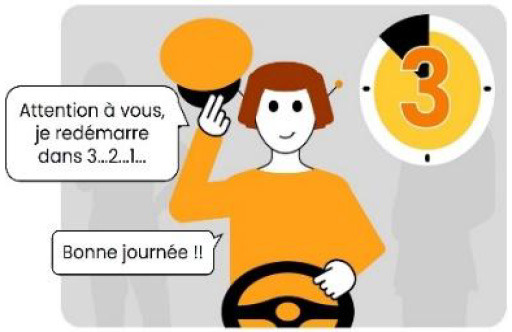
Starting	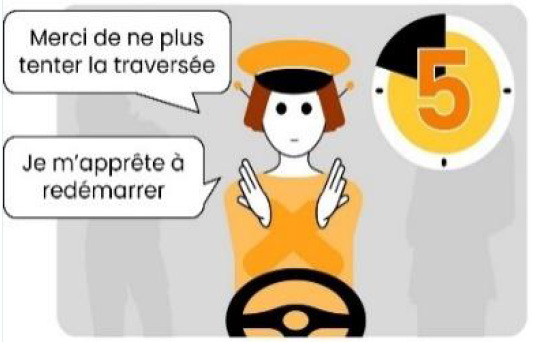	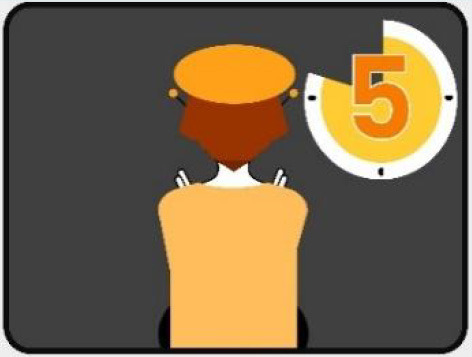
Driving	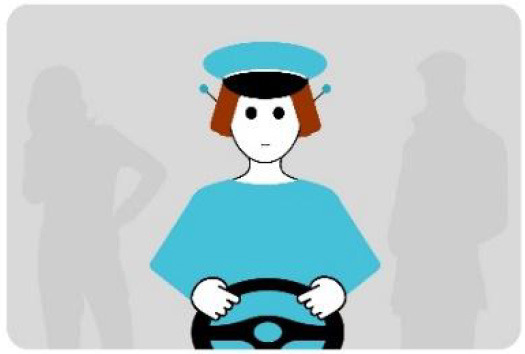	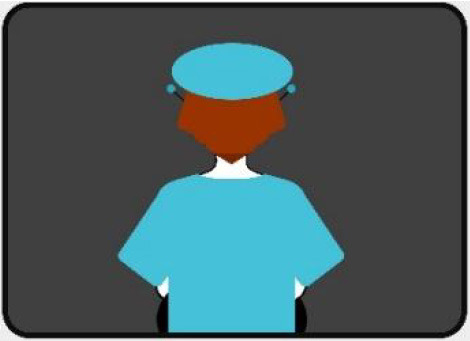
Pedestrian in sight	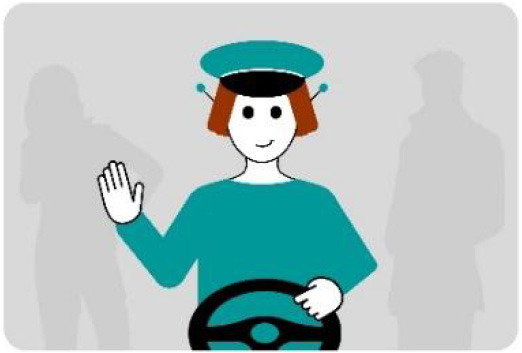	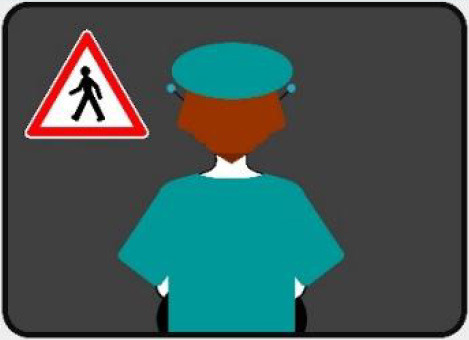
Safe crossing	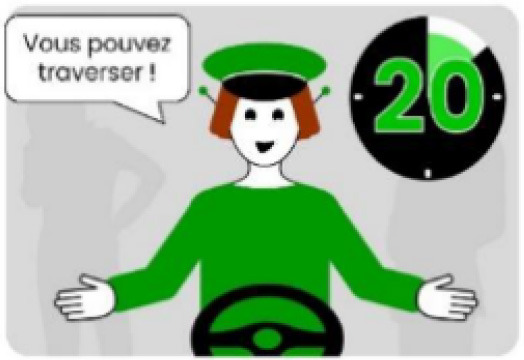	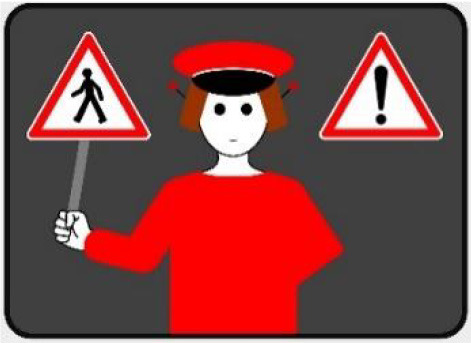
Unsafe crossing	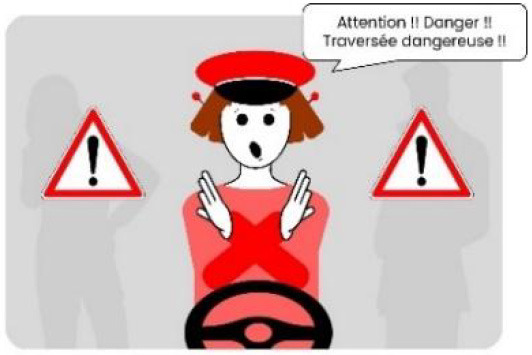	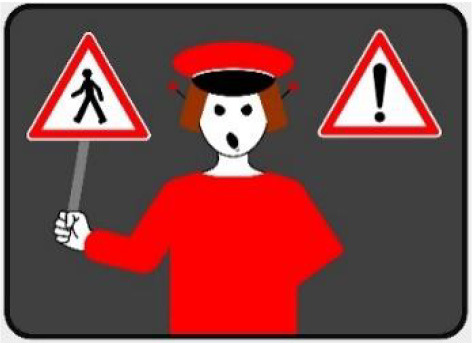

**Table 4:** Representations of messages displayed by the human–machine interfaces SIROCCO.

**Messages**	**General view**	**Traffic Lights**
(nominal situation)	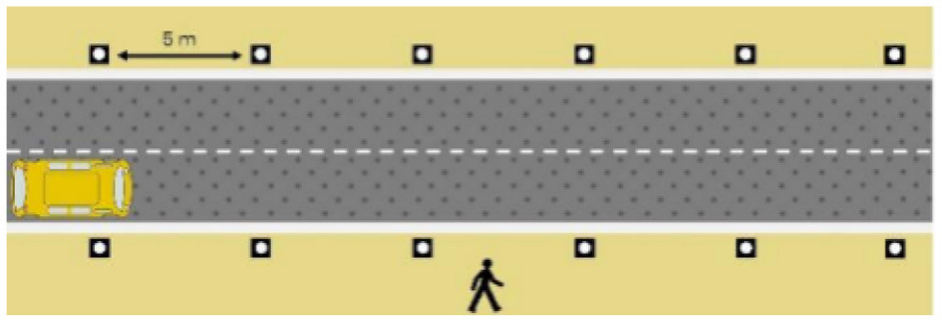	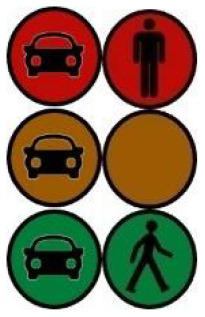
Consideration of the need to cross	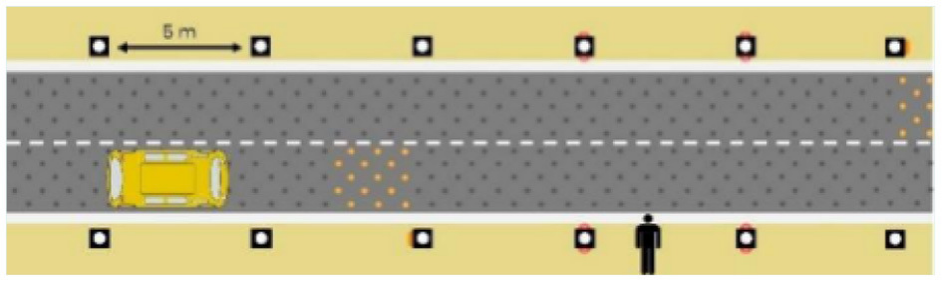	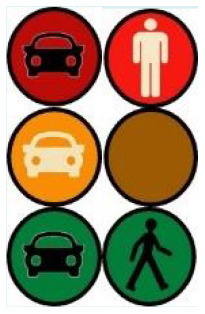
	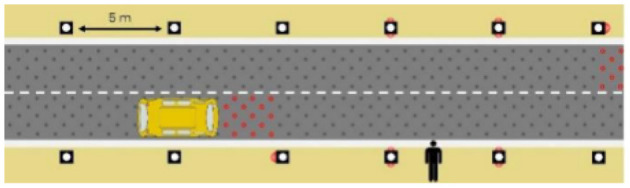	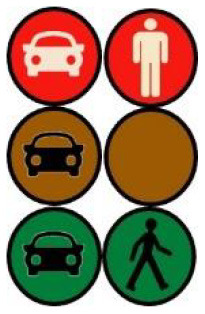
Safe crossing	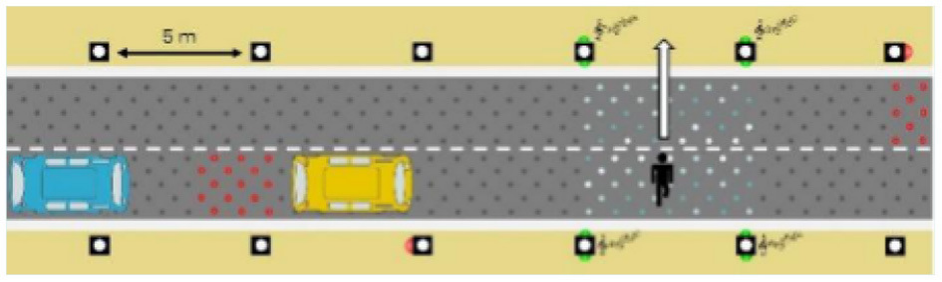	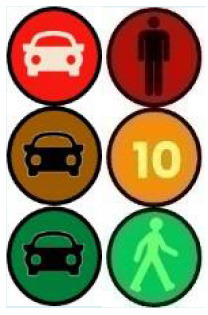
End of the crossing	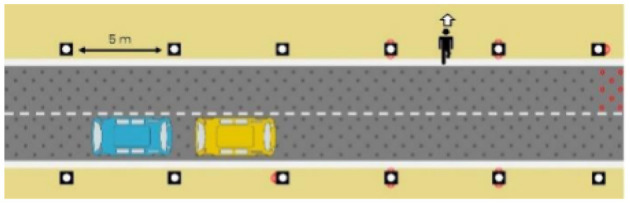	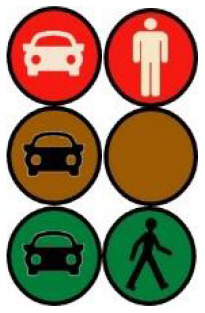
Return to nominal situation	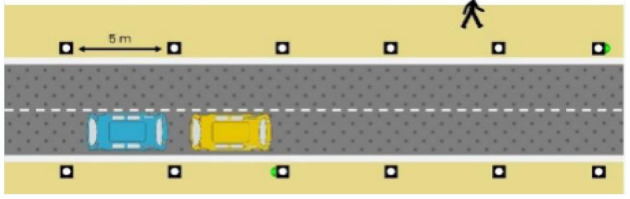	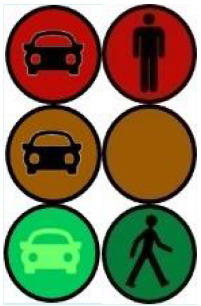
Unsafe crossing	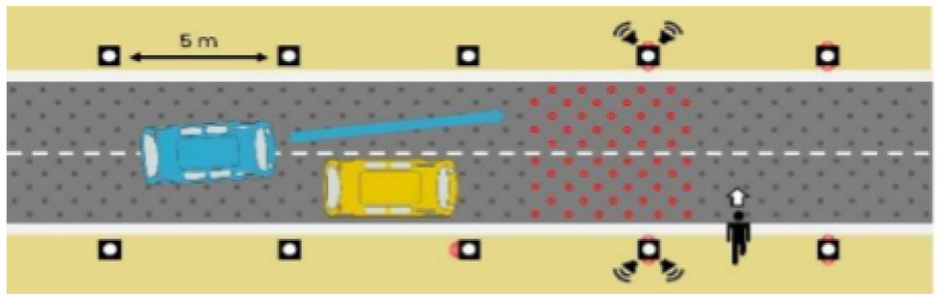	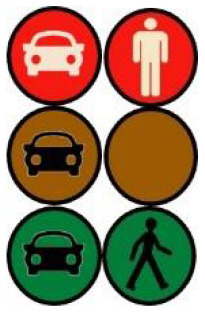

#### 2.3.2. Questionnaires

After each presentation video of AV and HMIs, the participants were evaluated through an online survey their feelings (i.e., trust, distrust, anticipation, uncertainty, usability, perceived safety, usefulness, attraction, and intention of use) and two types of crossing behaviors: normed and non-normed. The normed crossing referred to a crossing on the pedestrian crossing. Non-normed crossing behaviors referred to the willingness to cross on a pedestrian crossing while looking at their phone or chatting with friends (i.e., dual-tasking), or else, to cross aside from the pedestrian crossing.

As all the participants have already experienced crossing in front of a conventional vehicle, we have not considered the interaction with a CV as the main experimental condition of our research. To compare the pedestrian's experience with AV and CV, we assess the trust level and the willingness to cross also in the context of interaction with a CV. Age, gender, and habits of anticipation for street crossing were also collected. Each measure was made on a 6-point Likert scale (from 1 = “very low” to 6 = “very high”). Nevertheless, general satisfaction and intention to use of HMIs were measured on a 10-point scale (1 = “very low” to 10 = “very high”) to improve the possible discrimination (see Dawes, 2008) between the three HMIs in the context of a more global assessment by these two measures. The term “device” was used instead of “interface” to ease participants' understanding. The Table 5 resumes the different variables used in the questionnaire.

**Table 5:** Used questionnaire of the present study.

**Dependent variables**	**Question statements**
Street crossing habit	Most of the time, I start crossing the street before the vehicle has completely stopped.
Intention of use	I would like [Alfy – BOLD – Sirocco] to be implemented when automated vehicles arrive in the future.
Ranking of perceived safety intake	Which device will most increase your safety when crossing in front of an automated vehicle?
Ranking of trust intake	Which device will most increase your trust when crossing in front of an automated vehicle?
Ranking of satisfaction	Which device do you prefer? Rank them in order of preference.
Use case n°1(i.e., safe street crossing situation), question statements: If I was on the side of the road when an automated vehicle (equipped with Alfy or BOLD) arrives…(OR) If I was on the side of a connected road, the Sirocco, when an automated vehicle arrives…	
Utilisability	It will be easy to know if I can cross or not.
Perceived usefulness	I perceive the usefulness (of the automation of driving/of the device) for crossing the street.
Satisfaction	I like the device.
Anticipation of action	I will cross before it even stops completely.
Perceived safety	I will feel safe during the crossing.
Trust	I will trust that he will stop to let me cross.
Trust vs. CV	I will have more trust in it than in a vehicle with a driver
Uncertainty	I will hesitate before crossing.
Distrust	I will be suspicious that it will stop to let me cross.
Normed crossing behavior	I will cross on the pedestrian crossing.[indicated by Sirocco]
Non-normed crossing behavior	I will cross even if there is no pedestrian crossing. I will cross even if pedestrians' lights on the Sirocco are red.
Dual-task crossing behavior	I will cross while doing other things like looking at my phone or chatting.
Willingness to cross vs. CV	I will rather cross in front of it than in front of a vehicle (with a driver/without the HMI).
Use case n°2(i.e., dangerous street crossing situation), question statements: This indication of danger given by (Alfy/BOLD/Sirocco)…	
Utilisability	…will let me understand if I should cross or not.
Perceived usefulness	…will be useful.
Satisfaction	…appeals to me.
Perceived safety	…will give me a general feeling of safety when crossing in front of the automated vehicle.
Trust	…will give me a general feeling of trust when crossing in front of the automated vehicle.
Willingness to cross	…will make me want to cross.

### 2.4. Procedure

Participants have filled in the questionnaire online. They were presented with an introductory text informing them about the ethical processing of collected data and the purpose of the study, i.e., an evaluation of three interfaces aiming to facilitate pedestrians' interaction with an automated vehicle. They had to test their audio–visual material before going to the different parts of the questionnaire. First, data were collected about a crossing situation in front of a fully automated vehicle, then with the three communication systems in a counterbalanced order. At the end of each section evaluating an interface, they had to evaluate their levels of attractiveness as a percentage and whether they would like it to be implemented in future with AVs. At last, participants had to rank the three HMI in terms of trust, security, and attractiveness. The questionnaire ended with sociodemographic data including age and gender. The questionnaire was completed in December 2021 and lasted 25 min on average.

### 2.5. Data analysis

Responses were considered as continuous data, and mean values were compared using multivariate analyzes of variance (ANOVA of 3 to 5 factors) or by Student's t-test for group comparisons. Data were homogeneous but had non-normal distribution; however, given a large number of participants (*N* = 731), parametric tests were conducted. Multivariate Pearson's correlations were performed with trust and distrust variables. By using a table of occurrences, rankings were analyzed according to a draw without replacement.

## 3. Results

### 3.1. Trust and distrust in automation

**Figure 4:**
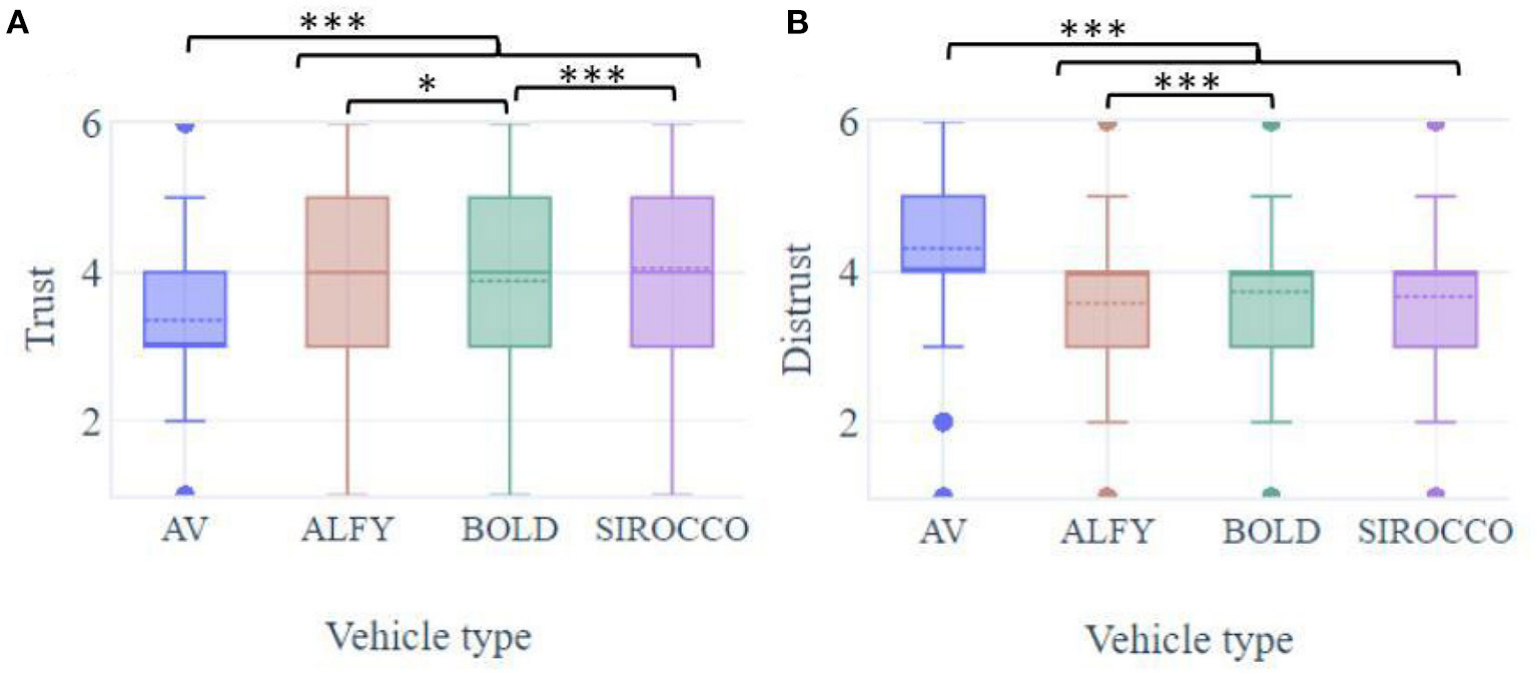
Boxplots of pedestrians' **(A)** trust and **(B)** distrust regarding the presence of HMIs during interaction with an automated vehicle. *p < 0.05 and ***p < 0.001.

Participants feeling of trust in a projected crossing situation in front of an AV were significantly influenced by the presence of HMIs [F(3.728) = 102, *p* < 0.001; see Figure 4A]. Self-reported trust levels were significantly higher with the HMIs (means out of 6; MVA = 3.36; SD = 1.4; MALFY = 4.0; SD = 1.26; MBOLD = 3.88; SD = 1.26; MSIROCCO = 4.05; SD = 1.26; *p* < 0.001 for all comparisons), especially with the Sirocco and Alfy compared to BOLD (*p* < 0.001; *p* < 0.05). Sirocco and Alfy induced trust levels that were not significantly different (*p* = ns). Analyzed according to a draw without replacement, the ranking showed that participants considered Sirocco as being the most reassuring HMI (40.6% of participants ranked it first), followed by BOLD and Alfy, the least reassuring during pedestrians' street crossing (35.7 and 36.0% of participants ranked them second and third).

The presence of HMIs induced a significant effect on projected distrust toward crossing in front of an AV [F(3,728) = 87.7, *p* < 0.001; Figure 4B]. Participants' distrust was significantly attenuated when HMIs were added to the crossing situation with AVs (MVA = 4.31; SD = 1.24; MALFY = 3.58; SD = 1.29; MBOLD = 3.74; SD = 1.20; MSIROCCO = 3.67; SD = 1.29; *p* < 0.001 for all comparisons). Compared to BOLD, Alfy significantly lowered the distrust feeling of participants (*p* < 0.001). It is interesting to note that there were no significant differences between the Sirocco and the other HMIs in distrust levels (*p* = ns).

### 3.2. Perceived safety

**Figure 5:**
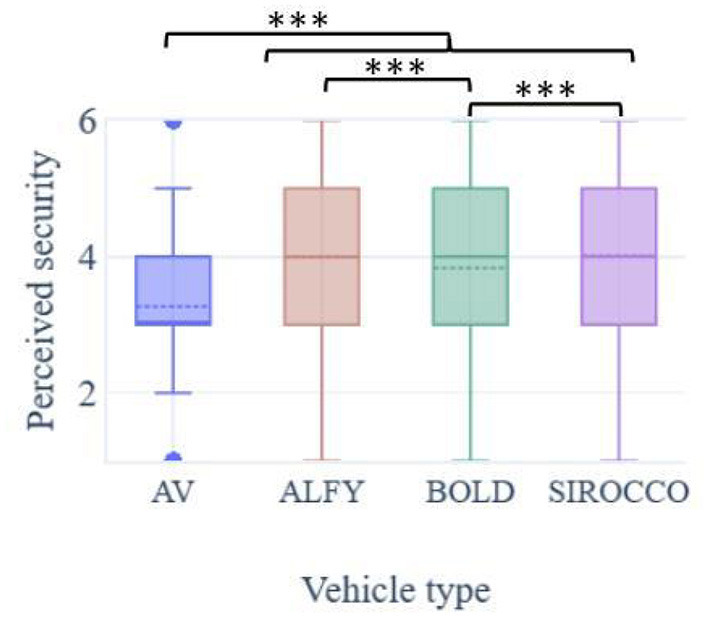
Pedestrians' perceived safety during crossing in front of an automated vehicle.***p < 0.001.

In general, the participant's sense of safety during a projected interaction with an AV was above average for all conditions but higher with HMIs (means out of 6; MVA = 3.27; SD = 1.24; MALFY = 3.99; SD = 1.27; MBOLD = 3.83; SD = 1.24; MSIROCCO = 4.02; SD = 1.27). The presence of HMIs improved significantly participants' feeling of safety during crossing in front of an AV [F(3.728) = 130, *p* < 0.001; *p* < 0.001 for all comparisons; Figure 5]. Sirocco induced a significantly higher security level compared to the other HMIs, while Alfy produced a more secure feeling than BOLD (*p* < 0.001 for all comparisons). When participants had to rank the HMIs according to their safety feeling in a road crossing situation with an AV, they designated Sirocco as the safest, followed by BOLD and Alfy. Analyzed according to a draw without replacement, the ranking showed that participants considered Sirocco as being the safest HMI (47.2% of participants ranked it first), followed by BOLD and Alfy, as the least capable of bringing security during pedestrians street crossing (37.3 and 37.6% of participants ranked them second and third).

### 3.3. Anticipation and uncertainty

**Figure 6:**
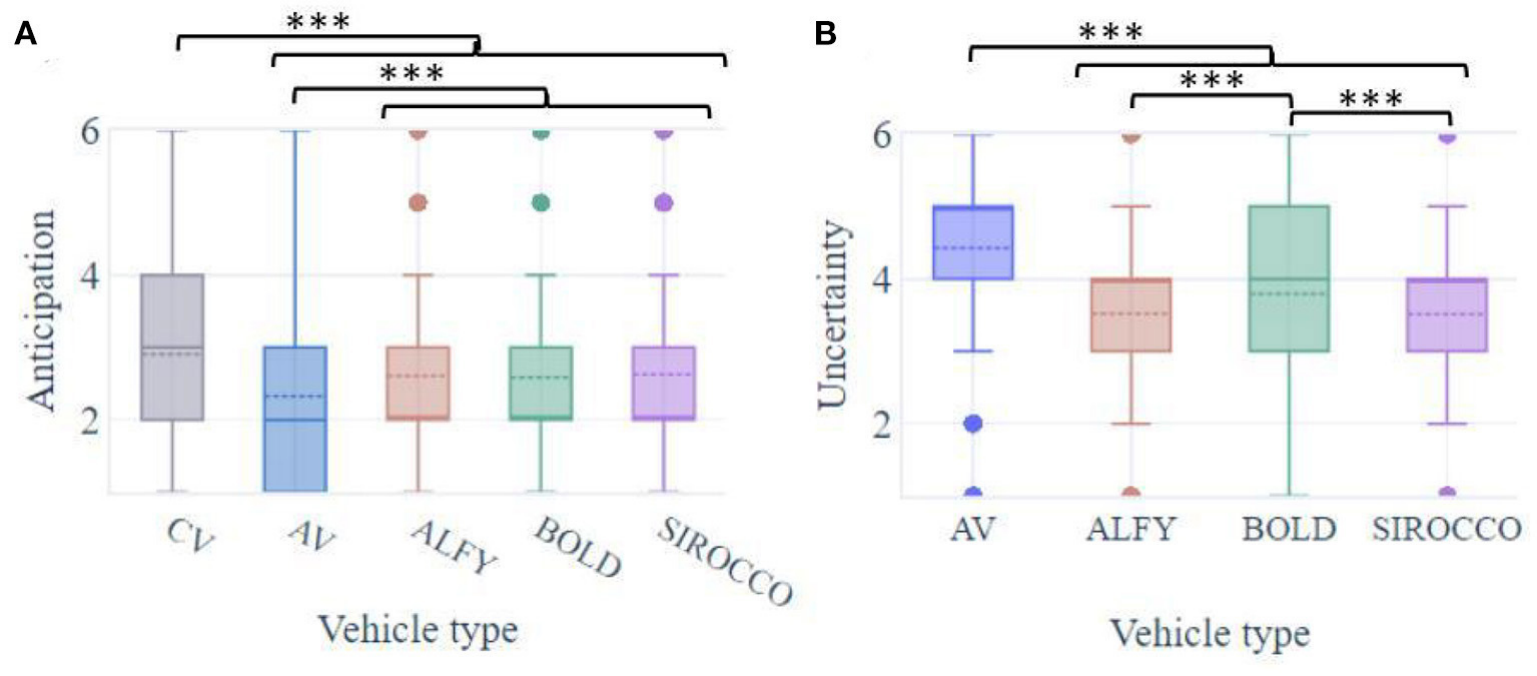
Boxplots of pedestrians' **(A)** anticipation and **(B)** uncertainty during interaction with an automated vehicle with HMIs. ***p < 0.001.

Two-thirds of respondents used to cross before the complete stop of the conventional vehicle (36.53%, mean CV = 2.91, SD = 1.43; Figure 6). In contrast, only 16.96% were willing to anticipate the crossing before the AV stop (MVA = 2.32, SD = 1.24), and 23.07% when the vehicle was equipped with IHMs (MALFY = 2.6; SD = 1.28; MBOLD = 2.59; SD = 1.26; MSIROCCO = 2.63; SD = 1.31; Alfy: 22.57%; BOLD: 22.44%; Sirocco: 24.21%; Mean of all HMIs = 2.61, SD = 1.10). A significant main effect of vehicle type (conventional, automated with, or without HMI) was found on participants' anticipation of the crossing [F(4.727) = 34.1, *p* < 0.001; Figure 6A]. *Post hoc* analyses showed that participants reported significantly greater anticipation of the crossing when encountering a conventional vehicle than an AV on its own or with HMIs (*p* < 0.001 for all comparisons). In addition, the presence of HMIs in a crossing situation with AV increased significantly anticipation (*p* < 0.001 for all comparisons), without any distinction of effect among them (*p* = ns).

The uncertainty of participants before the crossing was significantly affected by the tested conditions [F(3.728) = 139, *p* < 0.001; Figure 6B]. The presence of HMIs has significantly decreased the uncertainty of pedestrians during the crossing in front of an AV (MVA = 4.43; SD = 1.27; MALFY = 3.52; SD = 1.31; MBOLD = 3.79; SD = 1.25; MSIROCCO = 3.51; SD = 1.33; *p* < 0.001 for all comparisons). Especially, BOLD has significantly procured the most uncertainty in participants compared to Alfy and Sirocco (*p* < 0.001 for all comparisons) both latter had non-different levels (*p* = ns).

### 3.4. Road crossing behaviors

**Figure 7:**
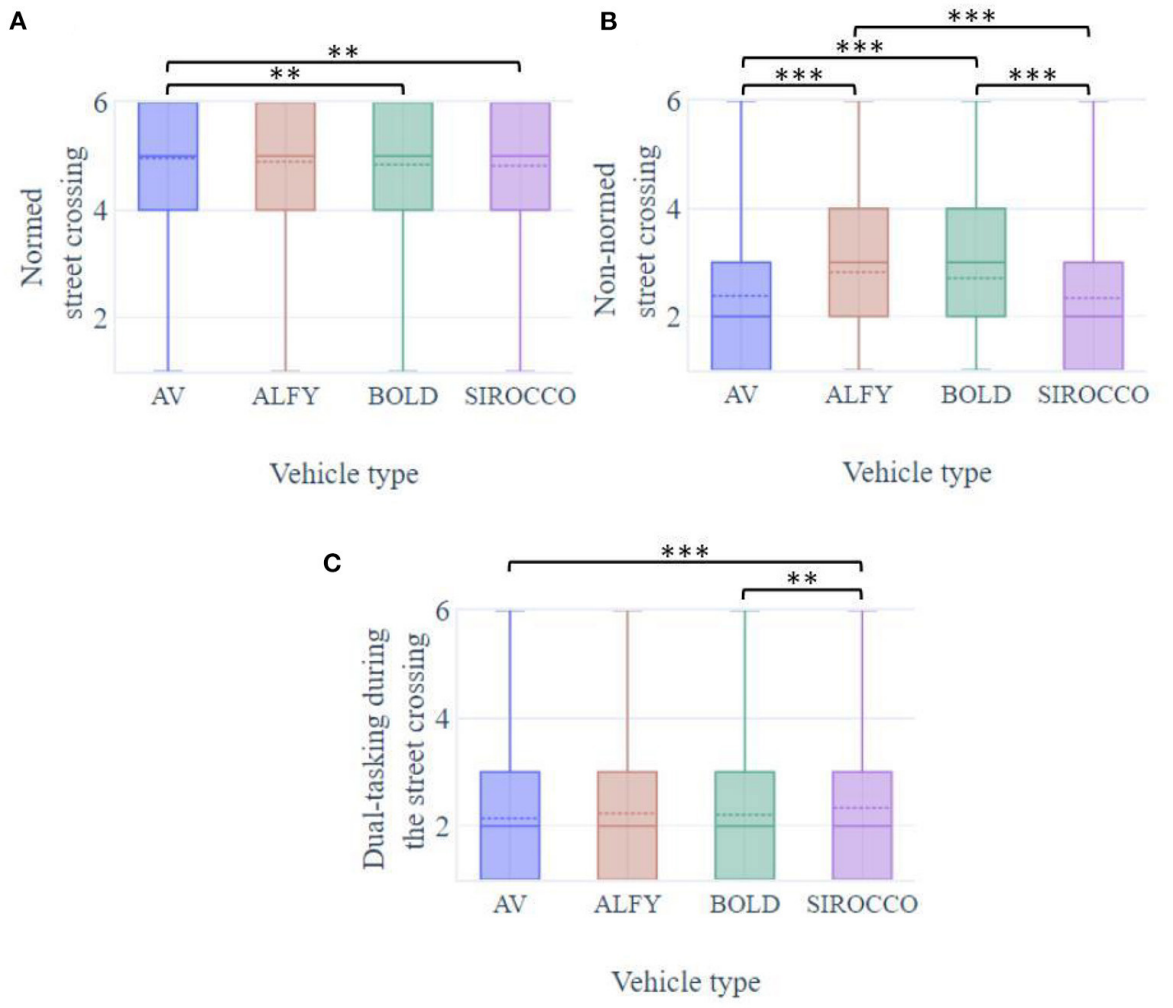
Boxplots of pedestrians' willingness to perform **(A)** normed crossing, **(B)** non-normed crossing, and **(C)** double task in front of AV with HMIs. **p < 0.01 and ***p < 0.001.

Results showed that the type of vehicle (conventional, automated with or without HMIs) had a significant effect on participants' crossing behaviors (Figure 7).

- For all conditions, the willingness to cross on the pedestrian crossing, i.e., normed crossing, reported by participants was high on average (MVA = 4.96; SD = 1.04; MALFY = 4.89; SD = 0.98; MBOLD = 4.84; SD = 0.98; MSIROCCO = 4.82; SD = 1.01); however, there was a significant effect of the presence of HMIs (F(3.728) = 4.93, *p* < 0.001; Figure 7A). Participants reported that they were less likely to cross if the AV was associated with BOLD or Sirocco (*p* = 0.011; *p* = 0.003). Other comparisons were not significant (*p* = ns).
- As expected, in the non-normed crossing (i.e., away from a pedestrian crossing or at red pedestrian lights on Sirocco), results suggested a lower level of willingness to cross (MVA = 2.38; SD = 1.27; MALFY = 2.82; SD = 1.31; MBOLD = 2.71; SD = 1.25; MSIROCCO = 2.34; SD = 1.26), though HMIs had significant effects [F(3.728) = 47.8, *p* < 0.001; Figure 7B]. An AV equipped with Alfy or BOLD induced significantly more willingness to cross than a street equipped with Sirocco (*p* < 0.001 for all comparisons). Reported levels between the two external HMI were not significantly different (*p* = ns).
- Globally, participants declared low willingness to perform a dual-task during the crossing in front of an AV (MVA = 2.14; SD = 1.22; MALFY = 2.23; SD = 1.25; MBOLD = 2.20; SD = 1.23; MSIROCCO = 2.33; SD = 1.31) but each HMI had a significantly different impact [F(3.728) = 8.38, *p* < 0.001; Figure 7C]. The willingness of dual-tasking was significantly more reported in the crossing conditions with Sirocco than with BOLD or an AV without an interface (*p* < 0.001; *p* = 0.007). Other conditions were not significantly distinct (*p* = ns).

### 3.5. Human–machine interfaces acceptance

**Figure 8:**
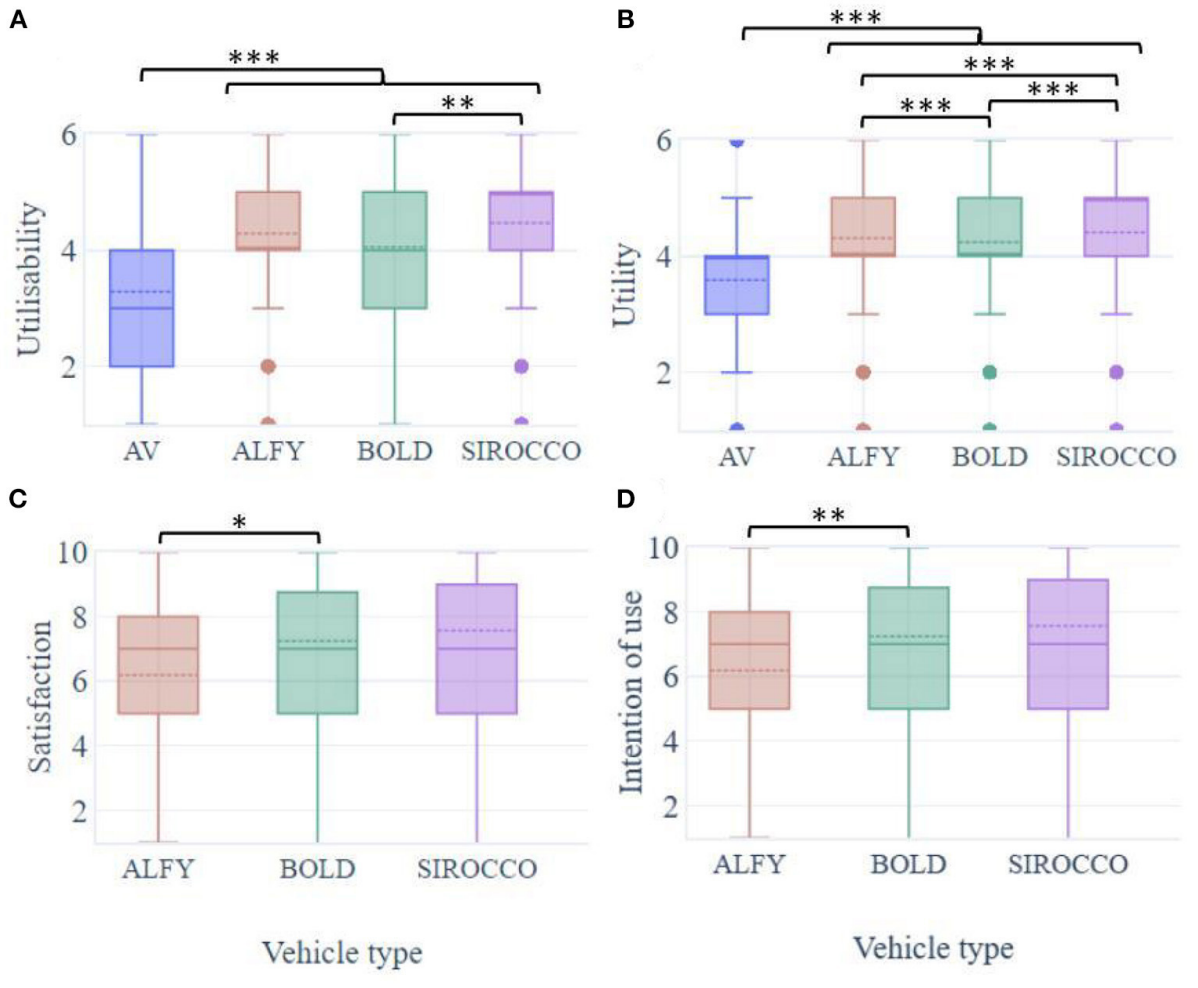
Boxplots of pedestrians' **(A)** perceived usefulness, **(B)** usability of the crossing situation, **(C)** satisfaction, and **(D)** intention of use regarding the HMIs. *p < 0.05, **p < 0.01, and ***p < 0.001.

Globally, participants found the HMIs useful (means out of 6: MAV = 3.59; SD = 1.31; MALFY = 4.31; SD = 1.19; MBOLD = 4.24; SD = 1.22; MSIROCCO = 4.41; SD = 1.26) and helping to understand (i.e., usability: MVA = 3.29; SD = 1.41; MALFY = 4.29; SD = 1.23; MBOLD = 4.05; SD = 1.24; MSIROCCO = 4.41; SD = 1.26) the crossing situation in front of an AV. Compared to a crossing situation with an AV, HMIs' presence has significantly influenced the perceived usefulness [F(3.728) = 106, *p* < 0.001; Figure 8A]. HMIs were rated useful to the road crossing in front of an AV (mean of HMIs = 4.32, SD = 0.99; *p* < 0.001 for all comparisons with the interfaceless condition). BOLD was perceived to be significantly less useful than the Sirocco (*p* = 0.007). Other comparisons with Alfy were not significant (*p* = ns). Usability, i.e., understanding of the crossing situation, was also influenced by HMIs [F(3.728) = 178 *p* < 0.001; Figure 8B] was facilitated by the HMIs (*p* < 0.001 for all comparisons). In the projected interaction with an AV, Sirocco was significantly more effective to enhance the participant's understanding of the crossing situation compared to Alfy and BOLD (*p* = 0.006, *p* < 0.001). Furthermore, the participants reported that Alfy was significantly more effective than BOLD to help them understand the road crossing interaction with an AV (*p* < 0.001).

In general, participants were satisfied with the HMIs (means out of 10; MALFY = 6.19, SD = 2.88; MBOLD = 6.43, SD = 2.78; MSIROCCO = 6.35, SD = 2.90; Figure 8C) and they wanted them to be integrated into the road crossing situation with AVs to use them (means out of 10; MALFY = 6.46, SD = 2.02; MBOLD = 6.76, SD = 2.94; MSIROCCO = 6.6, SD = 2.04; Figure 8D). The level of attractiveness and intention of use was significantly different depending on the HMIs [F(2.729) = 3.74, *p* = 0.024; F(3.728) = 4.33, *p* = 0.013]. Comparing the eHMIs, participants reported being significantly more attracted to and willing to use BOLD than Alfy (*p* = 0.021; *p* = 0.009). However, the results did not show any significant difference in comparison with the new infrastructure (*p* = ns). In the overall preference ranking, analyzed according to a draw without replacement, the Sirocco was the most popular interface (37.3% of participants ranked it first), ahead of BOLD and Alfy (39.0%, and 41.7% of participants ranked them in second and third).

### 3.6. Dangerous street crossing case

**Figure 9:**
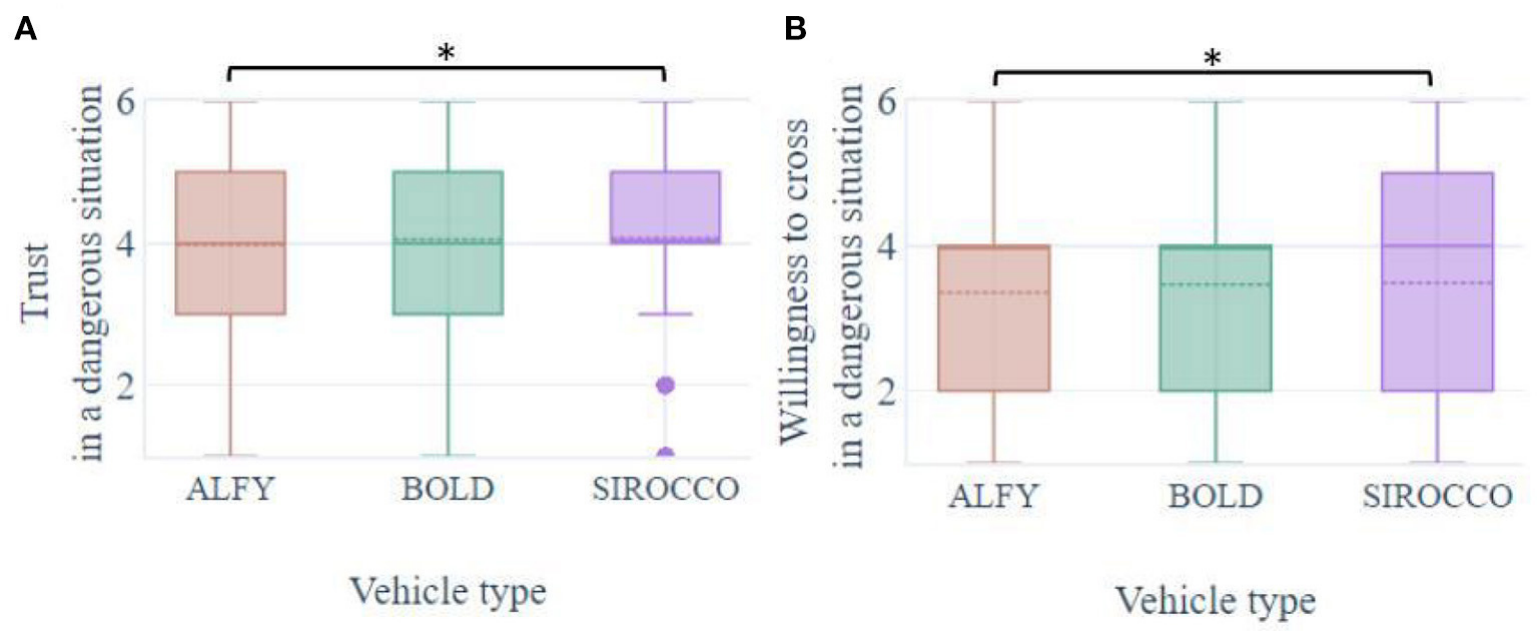
Boxplots of pedestrians' **(A)** trust and **(B)** willingness to cross in front of an automated vehicle with HMIs in a dangerous street crossing situation, i.e., non-standard use of HMIs. *p < 0.05.

During a dangerous crossing situation, where the HMIs advised against crossing, the participants projected themselves to have a high level of trust (means out of 6: MALFY = 3.97, SD = 1.26; MBOLD = 4.06, SD = 1.24; MSIROCCO = 4.08, SD = 1.26; Figure 9A). The willingness to cross of participants was abnormally high in the dangerous crossing situation while the HMI indicated not to cross (means out of 6: MALFY = 3.35, SD = 1.46; MBOLD = 3.46, SD = 1.45; MSIROCCO = 3.49, SD = 1.49). This means that on average, participants would cross despite the danger indications of the HMIs. Participants' trust, like their behaviors, was significantly influenced by the type of interfaces [F(3,728) = 84.6, *p* < 0.001; F(3,728) = 84.6, *p* < 0.001; Figure 9B]. In this dangerous context, Alfy induced significantly less trust and less willingness to cross the street compared to Sirocco (*p* = 0.035; *p* = 0.024). There was no significant difference for the other comparisons on trust and behavior (*p* = ns). Moreover, during the dangerous crossing situation, there was no significant difference between the interfaces on the other variables, namely usability, usefulness, attractiveness, and perceived safety (*p* = ns). However, the averages collected (means of all HMIs was >4) showed that the participants judged positively the interfaces without distinctions or messages:

- were useful (MALFY = 4.73, SD = 1.09; MBOLD = 4.67, SD = 1.04; MSIROCCO = 4.75, SD = 1.07),
- Allowed to know whether to cross or not (MALFY = 4.6, SD = 1.13; MBOLD = 4.56, SD = 1.13; MSIROCCO = 4.61, SD = 1.13),
- were appreciated (MALFY = 4.26, SD = 1.25; MBOLD = 4.21, SD = 1.22; MSIROCCO = 4.3, SD = 1.26) and,
- has enhanced feeling of safety (MALFY = 4.03, SD = 1.26; MBOLD = 4.08, SD = 1.25; MSIROCCO = 4.11, SD = 1.26).

### 3.7. Evaluation of hypotheses

The study supported all the hypotheses (Table 6). The results of this study supported that the presence of HMI specifically designed to promote trust would increase trust in the AV (H1a) and would lower pedestrians' distrust in street crossing conditions with an AV (H1b). Our results partially supported that an anthropomorphic HMI (i.e., ALFY) will increase pedestrians' trust to cross in front of an AV relatively more than an HMI designed with more conventional road signaling (i.e., BOLD; H2). Overall, pedestrians reported significantly more trust toward AV with the anthropomorphic HMI. H3a, which suggested that a communicative road infrastructure will be more efficient to increase pedestrians' trust than an HMI integrated into the automated vehicle, was partially supported. Additionally, we hypothesized that all the interfaces could impact the overall user's experience of street-crossing (H3b) and this was also partially supported by our results. In line with previous results, the pedestrian sense of security increased and uncertainty decreased when HMIs were associated with the AV but significantly less with BOLD than others compared to a crossing situation in front of an AV on its own. As well, pedestrian distrust when encountering an AV decreased with HMIs, especially, Alfy was more efficient than BOLD to defuse distrust feelings. Surprisingly, the results highlighted that participants were less willing to anticipate the crossing in front of an AV than a conventional vehicle. However, the use of HMIs reduced this effect. We also found that HMIs improved dangerous crossing behaviors (i.e., away from crosswalks or double-tasking). Especially, eHMIs induced more willingness to infract marked crossings. These behavioral results are evidence of pedestrians' overtrust in HMIs or AVs. Noted that, as the Sirocco substituted the road marking and provided a secure space for pedestrians, crossing behaviors on a pedestrian crosswalk become obsolete, and new behaviors can emerge. Furthermore, it is interesting to note that between eHMIs, we did not find any significant differences in pedestrians' road-crossing behaviors. Finally, we hypothesized (H4) that the different HMIs may influence differently the participants' trust and willingness to cross according to if they are confronted in a standard or in a non-standard use case (i.e., riskier, no nominal). This hypothesis was partially supported by our findings. First, results did not show significant differences between HMIs in perceived safety. Second, contrary to the standard use case, pedestrian trust was significantly higher with Sirocco than with Alfy but not significantly from the BOLD effects. Third, despite the indications of danger from the HMIs, the willingness to cross participants was abnormally high. Consistently with previous findings, this suggested an overtrust toward interfaces which provoked dangerous crossing behaviors.

**Table 6:** Summary of the hypothesis evaluation of the study.

**Hypothesis**	**Results**
H1 An HMI specifically designed to promote trust in AVs would enhance pedestrians' trust in a street crossing situation compared to an AV without HMI	Supported
H1.b Trust-based HMIs would decrease pedestrians' distrust in a street crossing situation compared to an AV without HMI.	Supported
H2 An anthropomorphic HMI (i.e., ALFY) will increase pedestrians' trust to cross in front of an AV relatively more than an HMI designed with more conventional road signaling (i.e., BOLD)	Partially supported
H3 A communicative road infrastructure will be more efficient to increase pedestrians' trust than an HMI integrated into the automated vehicle	Partially supported
H3.b All the user's experience of street-crossing that could be impacted by these HMIs	Partially supported
H4 Depending on the HMI, participants' trust and willingness to cross may also be different if they are confronted in a standard or a non-standard situation (i.e., more risky, no nominal).	Partially supported

Overall findings (i.e., in both use cases) reflected the HMIs participants' evaluation. HMIs were perceived as useful, satisfying, and improving understanding of the crossing situation when interacting with AV. Even if participants preferred BOLD, ALFY seemed to have a safer and more trusting effect over pedestrians during street crossing situations with an AV.

## 4. Discussion

The originality of these studies relied on the investigation of the effects of different ways of human–machine communication to strengthen pedestrians' trust and road crossing behaviors when interacting with automated vehicles: from a new infrastructure, a conventional road signaling, or even anthropomorphic features on the vehicle. Based on an online questionnaire filled in by 731 respondents, we highlighted the importance of road infrastructure and anthropomorphic information on the automated vehicle for pedestrian trust and safe behaviors.

### 4.1. Designing for road users' trust

To ensure safe and effective interaction with technology, consideration of the human role in its design is paramount (Lee et al., 2015). Abuse, misuse, or non-use of technology most often reflects an inadequate level of user trust in the machine (Muir, 1994); yet meeting users' needs (Florentine et al., 2015) or even improving their trust is rarely the goal of designers of technology tools (Bindewald et al., 2017). In contrast to the more traditional design process of HMIs, our user-centric approach focused on its trust by including the socio-relational and affective context of the human–machine interaction. Influencing trust allows us to act globally on the interaction with technology: modulating the whole of human activity, determined by internal, environmental, and organizational dimensions. This study highlighted that the overall road crossing experience with AV, including feelings of trust, safety, and appropriate behaviors of pedestrians, was improved with our HMIs compared to an interfaceless condition. This is in line with Lau et al. (2022) which showed that the use of eHMI communication improved the willingness to cross, trust, and safety feelings during the crossing in front of an AV. More broadly, this study validated the need for road information exchange to improve pedestrian–vehicle interaction (Matthews et al., 2017; Rouchitsas and Alm, 2019; Dommes et al., 2021).

### 4.2. Anthropomorphism or conventional road signs for in-vehicle interfaces

The eHMI BOLD provided crossing information through LED strips and pictograms using current road signaling codes and symbols (e.g., green/orange/red, pedestrian pictogram) to help pedestrians in their decisions and improve their trust during interaction with the AV. Consistent with previous research (Rouchitsas and Alm, 2019; He et al., 2021), participants were more attracted and wanted to use the LED eHMI (i.e., BOLD). However, BOLD produced significantly less trust and a globally more unsafe street crossing experience for pedestrians in front of an AV compared to the two other HMIs. BOLD, like the majority of eHMI prototypes in the literature (Dey et al., 2020), had the peculiarity of being entirely designed on the logic of road signage responding only to pragmatic decision support needs, while the other interfaces tested in this research (i.e., Alfy and Sirocco) relied on the hedonic needs of belonging and safety. This negative effect of eHMIs has already been spotted (Kaleefathullah et al., 2020) and is a warning toward designers of this type of HMI based solely on conventional road information. Holländer et al. (2019) stated that overtrust in this LED eHMI could lead to a misinterpretation of the AV's intentions, and lead to harmful situations. In contrast, this study highlighted the effect of socio-affective communication of drivers on pedestrians' feelings and street crossing behaviors. In comparison with the eHMI BOLD, the anthropomorphic communication system Alfy on the AV was significantly more efficient to induce pedestrians' trust and safe crossing behavior. Two conclusions can be made: either this result is evidence of a need for BOLD redesign (e.g., increasing the intelligibility of messages) or the integration of a hedonic, emotional, and social component to the human–machine interfaces of AVs is necessary to facilitate pedestrian decision-making. Either way, these findings are proof of evidence that current external human–machine interfaces (Dey et al., 2020) do not meet users' needs (Florentine et al., 2015), and further research is necessary to ensure safe interaction between pedestrians and automated vehicles.

In this study, the eHMI Alfy was designed to resemble a professional driver with facial expressions, voice intonations, and conventional body language such as hand gestures to greet. The novel contribution of this study is to compare the effectiveness of an eHMI communicating through body and verbal language with an eHMI mimicking conventional signage on the crossing activity of pedestrians in front of an AV. In other words, we compared the effect of socio-affective information vs. pragmatic “street crossing-focused” information on pedestrians' crossing activity at an AV. When pedestrians projected themselves into the situation of crossing in front of an AV, they felt significantly more safe and trustful while feeling less distrust in the presence of Alfy rather than BOLD. This effect of anthropomorphism in increasing trust in the AV aligns with observations from the literature in the field of human–machine interaction (e.g., Kraus et al., 2009; Verberne et al., 2015; Niu et al., 2018). It would seem that body language and verbal language are promising avenues for improving pedestrians' feelings, including trust, during their future interactions with the AV.

In terms of behavior, in contrast to Alfy, BOLD decreased the willingness of pedestrians to cross at pedestrian crossings compared to an AV without eHMI. In the dangerous crossing situation, pedestrians were more likely to project themselves to follow Alfy's indications (i.e., not to cross) rather than Sirocco. Being at different locations in the street crossing situation (i.e., one on the vehicle and the other in the environment), these two interfaces are limited in their comparisons. These results could be an argument in line with previous research (Madhavan and Wiegmann, 2007) highlighting the fact that the anthropomorphism of HMIs would result in more socially acceptable responses from the user. In our case, the anthropomorphism of the interface would have resulted in more compliant pedestrian behavior toward road norms (i.e., infrastructure) and information given by the Alfy eHMI compared to an eHMI without human features. However, this positive effect of anthropomorphism was not confirmed in all the norm violation situations tested. Indiscriminately, the in-vehicle devices (i.e., Alfy and BOLD) did not decrease dual-tasking behavior and significantly increased crossing behaviors outside of pedestrian crossings compared to an AV without the device. These mixed results can be explained by the levels of the measures collected, which were already low for non-normed crossing behaviors (i.e., double-tasking and crossing outside of a pedestrian crossing) and high for normed crossing behaviors (i.e., crossing in a pedestrian crossing), which limits the scope for evolution. Furthermore, these results are limited by the social desirability effect of the participants which was, in our case, to show their conformity to the norms of the road. However, these results need to be further investigated, as the behavioral results are sometimes contradictory (i.e., on normed vs. non-normed crossing).

### 4.3. Road infrastructure potential on pedestrians–automated vehicle interaction

Being located in a space open to all types of users, in case of error, failure, or even hacking, the AV could be a source of serious incidents or even accidents. The perception of safety is extremely important to initiate the user's trust that the technology will perform the expected task (Hoffman et al., 2006). It should be remembered that the road environment has been designed for the fluidity of vehicle traffic (Emanuel, 2021), making it a hostile space from the pedestrian's perspective. We designed the Sirocco in an environment of minimally connected vehicles. As soon as the pedestrian wanted to cross, through a traffic light system, the connected vehicles had to comply with his need as a priority. The basic assumption of the Sirocco concept was that it would be able to stop all vehicles detected upstream of the interaction point, systematically giving the pedestrian priority (and making it clear that he had priority and was safe). In our study, Sirocco was the favorite concept of the respondents (37% of participants), designated as the one that most increased trust (41% of participants), and safety (47% of participants) of pedestrians during future crossings in front of automated vehicles. Sirocco was always followed in the rankings by BOLD and then Alfy. Similarly, when participants projected themselves interacting with a VA in a Sirocco-equipped street, their overall trust was the highest of the three interfaces, compared to a conventional vehicle. Sirocco is an augmented version of the conventional crossing situation combined with elements known to increase pedestrians' trust, i.e., a pedestrian crossing and traffic lights (Cœugnet et al., 2019; Dommes et al., 2021). However, in the dangerous situation, pedestrians did not comply with Sirocco's indications (especially in comparison with Alfy). Furthermore, pedestrians projected themselves to dual-tasking, such as chatting or telephoning, while crossing with Sirocco, especially in comparison with BOLD. In other words, pedestrians perceived such a high level of trust that they would willingly delegate their safety management and crossing situation assessment tasks to the automated systems to be able to engage in other activities. This is consistent with the study of Sheridan (2000, p 142) showing that the purpose of automating systems is to “relieve humans of the need for situational awareness and the preparation for action based on that awareness”. Further investigations need to be conducted to better understand the implication of this new use of the road if it was equipped with such a device.

### 4.4. Limitations

First, France as a study context limited certain aspects of this study. Trust in an automated system is influenced by the temporality of errors perceived by the user (Hoff and Bashir, 2015). When a failure occurs early, it impacts user confidence more than an error that occurs later, suggesting the importance of the first impression left by the automated system. Due to more flexible standards and laws in the United States on AV experimentation compared to France, accidents that have occurred with AVs (in the United States), widely publicized (Rice, 2019), have affected the representations and Frenchs' trust in automation (Ah-Tchine, 2020). However, trust in automation seemed to be rebuilt as experiments are deployed in France. Thus, the results of this study are limited to the French population and similar cross-cultural research should be conducted. Second in this study, participants participated in the online survey from their computers. Therefore, several aspects of the experimental setup, e.g., video quality or monitor size, could not be controlled. Although we made sure that participants were watching carefully the HMIs presentation by testing them on non-related words (e.g., Christmas, turtle, and guitar) incorporated into videos, the experimental setting may have had an impact on the internal validity of this study. As the street crossing activity, trust is a mix of explicit and implicit attitudes. Thus, individuals who are non-expert or poorly trained in producing metacognitive reflections would be restricted or even unable to formulate the consequences of their implicit attitudes involved in their feelings of trust (Merritt et al., 2013) or to express their actions and reflections concerning street crossing (Zeedyk and Kelly, 2003). In addition, because participants did not interact with the AV or the HMIs, they were unable to evaluate properly how well these items would perform solely on mental projections. Tests in a natural environment could emphasize new usages of HMIs (Métayer and Coeugnet, 2021). If this study focused primarily on subjective measurements to investigate pedestrians' interaction with different HMIs and AVs, objective measures need to be addressed in future research (Lagstrom and Malmsten Lundgren, 2015; Beggiato et al., 2017; Hensch et al., 2021).

## 5. Conclusion and future studies

Trust help to overcome the cognitive complexity that people face in managing increasingly sophisticated automation (Lee and See, 2004). Thus, trust could be the key to securing and maintaining a human–machine relationship as we have highlighted with the Sirocco scenario. A trust-centered approach would, therefore, be a way to act on the whole user experience when interacting with technology. In addition, being designed to improve users' trust also entails risks. To ensure the performance of the human–machine interaction, it is crucial to appropriately calibrate the level of trust in the automated system (Muir, 1987; Wickens et al., 2000; Sheridan et al., 2002). Consistently with previous findings (Kaleefathullah et al., 2020), this study suggested an overtrust toward automation which could provoke dangerous crossing behaviors. In the case of overtrust in the automated system of one's vehicle, in the event of a failure, the user would lose the ability to guarantee his or her safety, which has led to fatal accidents (Rice, 2019). According to Marsh and Dibben (2005), distrust could be a means of regulating a situation of overtrust. In reality, an appropriate level of trust is a balance between the expectations of the user and the real capacities of automation (Muir, 1987; Lee and Moray, 1994). Thus, to avoid the future scenario in which pedestrians will have an overly high level of trust in AVs, we recommend the introduction of elements of distrust in automated technologies such as information about their technical limits. In other words, introducing uncertainty about the capacities of automation would lower users' expectations but lead to a safer human–machine interaction, when real technical capacity met perceived abilities. Another means would be favored by HMIs which cannot guarantee the complete safety of pedestrians, such as BOLD or AFLY. We thus agreed with Lee and See (2004) who argued that the main goal of human–machine research is to make systems highly automated, but not excessively “trustworthy.” Ultimately, trust would, therefore, be a relevant means to design, improve and maintain a human–machine interaction situation (Hjemly and Alsos, 2021). This study encourages future research to pursue the collection of needs and the design of users' trust-oriented artifacts to enrich the knowledge of the field of interaction with future technologies, both in the context of road users, but also in other contexts.

## Data availability statement

The raw data supporting the conclusions of this article will be made available by the authors, without undue reservation.

## Ethics statement

Ethical review and approval was not required for the study on human participants in accordance with the local legislation and institutional requirements. The patients/participants provided their written informed consent to participate in this study.

## Author contributions

FB: conceptualization, methodology, data collection, statistical analysis, writing the original draft, editing of sections, and revision. SC: conceptualization, methodology, editing of sections, revision, and supervision. EB: conceptualization and supervision. All authors approved the submitted version of the manuscript.
